# Local sequence and sequencing depth dependent accuracy of RNA-seq reads

**DOI:** 10.1186/s12859-017-1780-z

**Published:** 2017-08-09

**Authors:** Guoshuai Cai, Shoudan Liang, Xiaofeng Zheng, Feifei Xiao

**Affiliations:** 1 Department of Molecular and Systems Biology, Geisel School of Medicine at Dartmouth, Hanover, NH USA; 2 Department of Environmental Health Sciences, Arnold School of Public Health, University of South Carolina, Columbia, SC USA; 3 Department of Bioinformatics and Computational Biology, The University of Texas MD Anderson Cancer Center, Houston, TX USA; 4 Department of Epidemiology and Biostatistics, Arnold School of Public Health, University of South Carolina, Columbia, SC USA

**Keywords:** RNA-seq, Non-uniformity, Bias, Base-level modeling, Overdispersion, Beta-binomial, Differential expression analysis

## Abstract

**Background:**

Many biases and spurious effects are inherent in RNA-seq technology, resulting in a non-uniform distribution of sequencing read counts for each base position in a gene. Therefore, a base-level strategy is required to model the non-uniformity. Also, the properties of sequencing read counts can be leveraged to achieve a more precise estimation of the mean and variance of measurement.

**Results:**

In this study, we aimed to unveil the effects on RNA-seq accuracy from multiple factors and develop accurate modeling of RNA-seq reads in comparison. We found that the overdispersion rate decreased when sequencing depth increased on the base level. Moreover, the influence of local sequence(s) on the overdispersion rate was notable but no longer significant after adjusting the effect from sequencing depth. Based on these findings, we propose a desirable beta-binomial model with a dynamic overdispersion rate on the base-level proportion of sequencing read counts from two samples.

**Conclusions:**

The current study provides thorough insights into the impact of overdispersion at the position level and especially into its relationship with sequencing depth, local sequence, and preparation protocol. These properties of RNA-seq will aid in improvement of the quality control procedure and development of statistical methods for RNA-seq downstream analyses.

**Electronic supplementary material:**

The online version of this article (doi:10.1186/s12859-017-1780-z) contains supplementary material, which is available to authorized users.

## Background

Today, RNA-seq is a common technique for surveying RNA expression. Because sequencing read counts from individuals often show dispersion of measurements significantly larger than that given by Poisson distribution, fine modeling on this so-called *overdispersion* is required for RNA-seq data analysis [1, 2]. Negative binomial based distributions have been used by edgeR, DESeq/DESeq2, baySeq, and other methods to model overdispersed RNA-seq data for differential expression (DE) analysis [1–5]. Alternatively, beta-binomial distribution based methods have been proposed [6, 7]. However, these methods are still under development for more accurate model fitting, due to the elusive properties of RNA-seq read counts, especially from the aspect of dispersion. Dispersion of RNA-seq was strongly related to the sequencing depth [1], which was found to be critical to the power of detection of all expressed genes and differentially expressed genes between groups [8–10]. Previously, we investigated the variance of RNA-seq reads between samples with no biological difference, such as runs of different library preparations from the same sample, and found strong dependency between overdispersion and sequencing depth [7]. In the current study, we continued to study this scenario that samples have the identical genetic background, such as identifying differentially expressed genes in the same cell line with stimulation by a ligand.

RNA-seq data has many biases and effects which make developing accurate methods challenging [11–17]. Li et al. demonstrated the non-uniformity of RNA-seq reads by showing that the number of reads per nucleotide might vary by 100-fold across the same gene, which was caused by random hexamer priming bias in the nucleotide composition at the beginning of transcriptome sequencing reads [12, 13]. Therefore, a naive Poisson model, which assumes counts from all base positions are independently sampled from a Poisson distribution with a single rate proportional to the expression, is not appropriate. Several methods have been proposed to model local sequence related RNA-seq biases for transcript abundance estimation. Li et al. [13] proposed a method to predict variable rates based on local sequence and correct the non-uniformity, alpine [18] used a Poisson generalized linear model to model RNA-seq fragment sequence bias related to fragment GC content and GC stretches, and Salmon [19] provided a fast method with sample-specific bias models to capture fragment GC content bias and other effects. However, capturing the fluctuation at each base position among replicates, which is critical for precise RNA-seq data modeling and accurate differential expression analysis, is out of the research scopes of those tools. In this study, we aim to achieve an accurate modeling of RNA-seq reads with fluctuation estimation at each base position for comparison by taking random hexamer primer effect into consideration.

Given the same influence from the same local sequence of one particular gene, it is reasonable to assume that the mean number of sequencing reads on each base in one experimental condition is consistently proportional to that in another experimental condition. This assumption is supported by the observation in the study of Li et al. that the patterns of sequencing reads mapped to the same local sequences were highly consistent, even across different tissue types [13]. Therefore, we modeled the proportions of base-level coverage comparing two samples based on beta-binomial distribution, assuming the proportions have different dispersion but the same mean. Thus, high variable Poisson rates only enter the process indirectly through the dispersion which is advantageous in modeling. We previously observed decreasing gene-level overdispersion corresponding to increasing sequencing depth [7], which is expected to be true on base pair level as well. Therefore, local sequence composition and sequencing depth might be confounders in estimating overdispersion rate, and this remains unstudied. To investigate this confounding effect, we evaluated and compared three beta-binomial models: a full model with effects of both local sequence and sequencing depth and two reduced models with one of effect each.

Here, we focused on studying the dependency of overdispersion with sequencing depth and local primer sequence at base level. Large-scale consortium-based RNA-seq studies, such as ENCODE [20], MAQC [21], SEQC [22] and others, provide opportunities to investigate the properties of RNA-seq data and evaluate proposed methodologies. We estimated the base-level overdispersion rate of RNA-seq read count from ENCODE spike-in dataset which has a large sample size [23]. Also, we investigated the potential biases introduced by library preparation protocols including fragmentation and strand synthesis. We evaluated the fitting performance of the proposed beta-binomial models with a dynamic overdispersion rate and compared them to binomial model and beta-binomial model with a consistent overdispersion rate. In application to DE analysis, we compared our models with widely used methods including binomial test, *t* test, DESeq [1], edgeR [2] and limma-voom [24]. RNA-seq datasets related to the MAQC project with real-time PCR measurements were used in this comparison [25].

## Methods

### Datasets

Two datasets were used, the ENCODE spike-in dataset [23] and the MAQC dataset with real-time PCR data [25] (Table 1).

**Table 1:** Summary of the datasets used

ENCODE	ERCC	GSM758567 GSM758572 GSM758573 GSM758577 GSM765389 GSM765391 GSM765396 GSM765398 GSM767845 GSM767847 GSM767851 GSM767854 GSM767855 GSM767856			
MAQC	Brain	UHR library A	UHR library B	UHR library C	UHR Library D
	SRR037455	SRR037466	SRR037470	SRR037473	SRR037479
	SRR037456	SRR037467	SRR037471	SRR037474	
	SRR037457	SRR037468	SRR037472	SRR037475	
	SRR037458	SRR037469		SRR037476	

Training datasets were underlined

#### ENCODE dataset

Long NonPolyA RNAs from whole cells were measured in the ENCODE dataset. Two replicates from each of 14 human cell lines (Gm12878, Ag04450, Bj, Huvec, A549, H1hesc, Hepg2, K562, Hsmm, Mcf7, Nhlf, Sknshra, Nhek, and Helas3) were used in this study. Synthetic spike-in standards from the External RNA Control Consortium (ERCC) were sequenced along with human samples following the dUTP strand-specific sequencing protocol [23]. Two primers, mate1 and mate2, were used to distinguish specific strands. The sequencing reads from the ERCC libraries were mapped to the ERCC reference using Bowtie version 0.11.3 with parameters –v2 –m1 [26]. Gene-level abundances were estimated by counting uniquely mapped reads. We used samples (underlined in Table 1) with approximately the same total counts to estimate accurate dispersion between replicates by avoiding bias from sequencing depth. We truncated 76 nucleotides from the end of each gene as no count of 76 base-pair-long read was available in this region.

#### MAQC dataset

Bullard et al. measured two distinct MAQC reference samples, brain and UHR, using RNA-seq [25]. Four UHR libraries (A, B, C and D) and one brain library were prepared. RNAs were first fragmented and then converted into cDNAs using random hexamer priming approach. We used STAR [27] to align reads to the UCSC human genome hg19 assembly. Gene-level abundances were estimated by counting uniquely mapped reads in all exons. Additionally, 997 genes had previously been assayed by real-time PCR with high detection specificity and detection sensitivity, which can be used for validation of differential expression detection. We truncated 35 nucleotides from the end of each gene as no count of 35 base-pair-long read was available in this region.

### Estimation of Overdispersion rate *θ*_*ij*_ per base pair

Let *n*_*ij*_ and *m*_*ij*_ be the number of mapped reads starting at the *j*-th nucleotide of the *i*-th gene for the two samples in comparison, respectively. The probability mass function for the beta-binomial distribution is

1 $$ f\left({n}_{ij}|{\alpha}_{ij},{\beta}_{ij},{m}_{ij}\right)=\left(\begin{array}{c}{n}_{ij}+{m}_{ij}\\ {}{n}_{ij}\end{array}\right)\frac{B\left({n}_{ij}+{\alpha}_{ij},{m}_{ij}+{\beta}_{ij}\right)}{B\left({\alpha}_{ij}+{\beta}_{ij}\right)} $$

where *α*_*ij*_ and *β*_*ij*_ are two parameters of the beta-binomial distribution. The beta-binomial distribution can be represented using the following parameters: ${p}_{ij}=\frac{\alpha_{ij}}{\alpha_{ij}+{\beta}_{ij}}$ and ${\theta}_{ij}=\frac{1}{\alpha_{ij}+{\beta}_{ij}}$ for each *i* and *j*. Based on our assumption that the proportion of counts per base pair across a gene comparing two samples is a constant, *p*_*ij*_ is consistent for all positions on the *i*-th gene, as *p*_*i*_. Analytically, for the *i*-th gene with *J*_*i*_ base pairs, the true and unknown proportion *p*_*i*_ can be estimated as $\frac{\sum \limits_{j=1}^{J_i\ }{n}_{ij}}{\sum \limits_{j=1}^{J_i\ }{n}_{ij}+\sum \limits_{j=1}^{J_i\ }{m}_{ij}}$. Assuming most genes do not change, the neutral proportion of two samples *p*_*n*_ can be estimated from all (*J*_1_, *J*_2_,  … , *J*_*i*_,  … , *J*_*G*_) base pairs of all *G* genes as $\frac{\sum \limits_{i=1}^G\sum \limits_{j=1}^{J_i}{n}_{ij}}{\sum \limits_{i=1}^G\sum \limits_{j=1}^{J_i}{n}_{ij}+\sum \limits_{i=1}^G\sum \limits_{j=1}^{J_i}{m}_{ij}}.$ For any two replicates, the proportion of each gene should be equal to the neutral proportion, that *p*_*i*_ = *p*_*n*_. Based on the beta-binomial distribution, *θ*_*ij*_ can be estimated from the variance calculated from replicates as

2 $$ {\widehat{\theta}}_{ij}=\frac{\frac{1}{R}\sum \limits_{r=1}^R\left(\frac{\sigma_{p_{ij r}}}{p_{nr}\left(1-{p}_{nr}\right)}-\frac{1\ }{n_{ij r}+{m}_{ij r}}\right)}{1-\frac{1}{R}{\sum}_r^R\frac{\sigma_{p_{ij r}}}{p_{nr}\left(1-{p}_{nr}\right)}} $$

where *r* denotes the *r*-th pair among *R* total combination pairs of replicates and *p*_*nr*_ indicates the neutral proportion comparing the *r*-th pair. For the *j*-th nucleotide of the *i*-th gene from the *r*-th pair of replicates,${\sigma}_{p_{ijr}}$ indicates the variance of proportion, *n*_*ijr*_ and *m*_*ijr*_ indicate read counts mapped in the current pair of replicates. We estimated ${\sigma}_{p_{ijr}}$ from base-level read counts per replicate pair separately and estimated *θ*_*ij*_ according to formula (2).

### Base-level model

After reparametrizing by *p*_*i*_ and *θ*_*ij*_, the log-likelihood of the beta-binomial (Eq. 1) for the *i*-th gene with *J*_*i*_ base pairs was derived as

3 $$ \log \left({\mathcal{L}}_i\right)=\sum \limits_{j=1}^{J_i}\left[\sum \limits_{k=0}^{n_{ij}-1}\log \left({p}_i+k{\theta}_{ij}\right)+\sum \limits_{k=0}^{m_{ij}-1}\log \left(1-{p}_i+k{\theta}_{ij}\right)-\sum \limits_{k=0}^{n_{ij}+{m}_{ij}-1}\log \left(1+k{\theta}_{ij}\right)\right] $$

Previously, we proposed an efficient gene-level beta-binomial model for DE analysis with

$$ {\theta}_i=\frac{D_i}{{\left({n}_i+{m}_i\right)}^{\gamma }}, $$

in which *γ* represents the degree of dependency to sequencing depth [7]. *D*_*i*_ is a gene specific factor. In the current study, we assumed *D*_*i*_ to be consistent for all genes as *D* based on our observation. To achieve a better data fit, we propose a full model here, taking the local sequence around the first nucleotide of a read into consideration:

4 $$ {\theta}_{ij}=\frac{D{e}^{\left\{\sum \limits_{k=1}^K\sum \limits_{h\in \left\{A,\kern0.5em T,\kern0.5em C\right\}}{\beta}_{kh}I\left({b}_{ij k}=h\right)\right\}}}{{\left({n}_{ij}+{m}_{ij}\right)}^{\gamma }} $$

In this model, *K* is the length of the surrounding sequence around the *j*-th nucleotide of the *i*-th gene. We set *K* = 80 as suggested in the study of Li et al. [13] such that the surrounding sequence of 40 nucleotides before and 40 nucleotides after the *j*-th nucleotide was considered. Also, the indictor function *I*(*b*_*ijk*_ = *h*) is 1 when the *k*-th base pair is letter *h*, which is A, T, or C exclusively, and 0 otherwise. *D*, *β*_*kh*_, and *γ* are unknown parameters which require estimation. It is natural to assume *D* varies among sample pairs and thus pair-specific *D* will be estimated based on the determined *β*_*kh*_ and *γ*.

We took the logarithm of Eq. 4 and obtained the following formula that facilitates model fitting:

5 $$ \log \left({\theta}_{ij}\right)=\log (D)+\sum \limits_{k=1}^K\sum \limits_{h\in \left\{A,T,C\right\}}{\beta}_{kh}I\left({b}_{ij k}=h\right)+\gamma \log \left({n}_{ij}+{m}_{ij}\right) $$

Based on the observation of Wu et al. that the distribution of the logarithm of sample dispersion is approximately Gaussian distributed [28], we assumed log(*θ*_*ij*_) follows a Gaussian distribution and efficiently estimated these parameters using the linear least-squares approach in this study. In comparison to the sum of all the positions in all the genes, the parameter size in Eq 5, 240, is very small.

In order to investigate the confounding effect of the read depth and local primer sequence on the overdispersion rate, we further developed two reduced beta-binomial models: primer-free model (*β*_*kh*_ = 0) and depth-free model (*γ* = 0) in which the overdispersion rate was formulated as shown in the following Eqs. 6 and 7 respectively:

6 $$ \log \left({\theta}_{ij}\right)=\log (D)+\gamma \log \left({n}_{ij}+{m}_{ij}\right) $$

7 $$ \log \left({\theta}_{ij}\right)=\log (D)+\sum \limits_{k=1}^K\sum \limits_{h\in \left\{A,T,C\right\}}{\beta}_{kh}I\left({b}_{ij k}=h\right) $$

We refer to models shown in Eqs. 4, 5, 6, 7 as models with a dynamic dispersion rate. Alternatively, a beta-binomial model with a constant overdispersion rate was obtained when *γ* = 0 and *β*_*kh*_ = 0.

### Model fitting

To validate the dependency between local sequence, sequencing depth, and overdispersion, we set training datasets and test datasets. Training datasets shown in Table 1 were used to investigate the dependency of overdispersion, sequencing depth, and local sequence and determine the parameters of *γ* and *β*_*kh*_. Then, the captured dependency was borrowed to achieve better data fit and higher power of differential expression analysis on the test datasets.

- Estimation of *γ* and *β*_*kh*_
  1. Estimate ${\widehat{p}}_n=\frac{\sum \limits_{i=1}^G\sum \limits_{j=1}^{J_i}{n}_{ij}}{\sum \limits_{i=1}^G\sum \limits_{j=1}^{J_i}{n}_{ij}+\sum \limits_{i=1}^G\sum \limits_{j=1}^{J_i}{m}_{ij}}$ on the training set.
  2. Set *p*_*n*_ as a known parameter and obtain ${\widehat{\theta}}_{ij}$ according to Eq. 2. The least-squares estimation method is then applied to the full model (Eq. 5), the primer-free model (Eq. 6) and the depth-free model (Eq. 7) to estimate *γ* and *β*_*kh*_.
- Modeling test samples
  1. Initialize ${\widehat{p}}_i={\widehat{p}}_n$ in the beta-binomial model (Eq. 3) on the test set.
  2. Borrow the estimation of *γ* and *β*_*kh*_ from the training set for the full model and the primer-free model separately.
  3. Set *p*_*i*_ as a known parameter and maximize the beta-binomial log likelihood (Eq. 3) to estimate pair-specific *D*.
  4. Set *θ*_*ij*_ according to Eq. 4 as a known parameter and maximize the beta-binomial log likelihood to update ${\widehat{p}}_i$. This step is skipped when comparing replicates.
  5. Proceed to step 3 unless the deviance decreases less than 1%. This step is skipped when comparing replicates.

### Likelihood ratio test

According to the likelihood ratio test, −2 ln *ℒ*  (*p*_*n*_) + 2 ln *ℒ*  (*p*_*i*_) follows the *χ* ^2^ distribution with 1 degree of freedom, where *p*_*i*_ is the proportion for gene *i* and *p*_*n*_ is the neutral proportion. Equation 3 models the proportion of a pair of samples, which can be used to test samples without replicates by borrowing information from previously measured replicates. When replicates were available, we calculated the sum of their pairwise *χ* ^2^ scores comparing samples from two groups and obtained *p*-values with a summation of degrees of freedom.

### Model comparison

**Fig. 1:**
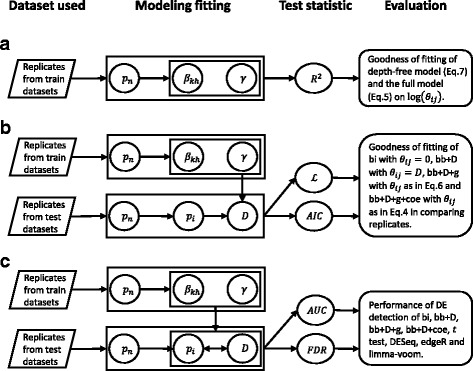
The strategy of model fitting and comparison

In this study, we evaluated the overall fitting of models. First, we evaluated the fitting of linear models shown in Eqs. 5 and 7 to study the confounding effect on overdispersion from sequencing depth and local sequence. Second, we compared models on data fitting in comparing the sequencing read counts from two replicates. Third, we assessed the performance of models in DE analysis. The strategies of comparison were shown in Fig. 1, including dataset usage, model fitting, test statistic, and evaluation purpose. Detailed methods for evaluating the models are as follows.

- Goodness of fit of the depth-free model (Eq. 7) and the full model (Eq. 5) on log (*θ*_*ij*_).
  We calculated the coefficient of determination *R* ^2^. We utilized the 5-fold cross validation strategy. Each of the training sets (shown in Table 1) were randomly split into five groups of equal size. In each round, we fit our model using four of these five groups, and then calculated *R* ^2^ on the remaining subset by the regression sum of squares divided by the total sum of squares. The process was repeated for 10 times and the overall cross-validation *R* ^2^ was determined by the mean.
- Goodness of fit of four models in comparing replicates, including the binomial model (bi) with *θ*_*ij*_ = 0, the beta-binomial model (bb + D) with *θ*_*ij*_ = *D*, the reduced primer-free model (bb + D + g) with *θ*_*ij*_ as in Equation 6, and the full model (bb + D + g + coe) with *θ*_*ij*_ as in Eq. 5.
*Likelihood value* We calculated the maximum likelihood values of pairwise comparisons of replicates to evaluate the goodness of fit. Proportion *p*_*i*_ was estimated as ${\widehat{p}}_n$ and fixed for all four models. Sequentially, other parameters were determined by our model fitting strategy (iterative fitting was skipped as *p*_*i*_ was fixed), and likelihood values were calculated based on estimated parameters. The *χ* ^2^ test was performed on *D* =  − 2 ln(ℒ_*nested*_) + 2 ln (ℒ), where ℒ and ℒ_*nested*_ are likelihoods for a model and its nested model, respectively.
*AIC* Akaike information criterion (AIC) is a measure of the relative goodness of fit of a statistical model. AIC was calculated by definition as 2*k* − 2 ln(ℒ), where *k* was the number of parameters and ℒ is the maximum-likelihood value. The overall AICs were determined by the mean of all AICs from pairwise replicates.
- Performance of DE detection of four models (bi, bb + D, bb + D + g, bb + D + coe) and widely used methods including *t* test, DESeq, edgeR and limma-voom. Evaluation was performed on MAQC dataset which has standard data for validation.
*AUC* The area under the receiver operating characteristic curve (AUC) was determined by the method described in our previous study [7].
*False housekeeping gene detections* To test the false discovery control ability, we assumed that housekeeping genes detected as differentially expressed genes at a given *p*-value were false discoveries. We compares the numbers of falsely discovered housekeeping genes given specific numbers of significantly differentially expressed genes. A list of 3804 housekeeping genes identified by Eisenberg and Levanon were used in this study [29].

### DE analysis methods in comparison

We compared our models with *t* test, binomial test, DESeq, edgeR and limma-voom on DE analysis. A two-tailed *t* test was performed on total counts normalized and logarithm transformed RNA-seq read counts. Four brain samples (SRR037455, SRR037456, SRR037457 and SRR037458) were compared to four UHR samples (SRR037469, SRR037472, SRR037476 and SRR037479) in the test datasets. The DE analyses in this study were performed using R version 3.2.5 and we applied packages “DESeq 1.22.1”, “edgeR 3.12.1” and “limma 3.26.9” to test the difference of sequencing read counts. “GLM” approach was used in DESeq and edgeR DE analysis. Normalization and model fitting were performed using the default parameters. When estimating the dispersions by DESeq, “local” fitType, “maximum” sharingMode and “pooled” estimation methods were used. All other parameters were set to the default in all DESeq, edgeR and limma-voom analyses. Functions of our proposed methods are available in the github repository (https://github.com/GuoshuaiCai/BBDG.git).

## Result

### Base-pair Overdispersion rate decreases with sequencing depth

**Fig. 2:**
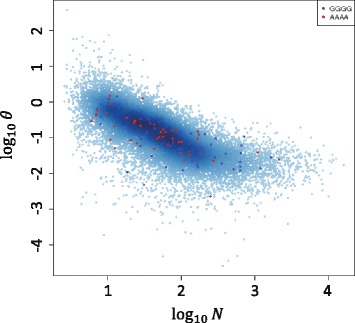
The relationship of overdispersion and sequencing depth. The base-level overdispersion rate of proportion *θ*_*ij*_ versus the mean tag counts in base 10 log scale. The *θ*_*ij*_ values were computed from replicates from the ENCODE spike-in training dataset. The *blue* and *red* points are for the positions with local sequences starting with GGGG and AAAA, respectively

We empirically investigated the effect of sequencing depth on the overdispersion rate of the measurement per base. Analyzing the ENCODE spike-in dataset, we calculated the variance of the proportion of the reads mapped to the *j*-th base pair of the *i*-th gene from replicates and then determined the overdispersion rate *θ*_*ij*_ (described in Methods). Figure 2 shows that the overdispersion rate was strongly inversely correlated with sequencing depth. That is, the overdispersion rate continually decreased as the sequencing depth increased without a sign of saturation. The correlation was sufficiently strong, causing the majority of the points to be concentrated along a line. This supported our assumption that all genes have consistent *D* and the proposed linear model shown by Equation 6. Moreover, local sequences starting with GGGG were found to have more sequencing reads and larger overdispersion than those starting with AAAA, indicating that hexamer priming might influence the overdispersion rate through affecting sequencing read counts. Therefore, local sequence and sequencing depth are not independent from each other and might be confounders.

### Sequencing procedure introduces extra noise

**Fig. 3:**
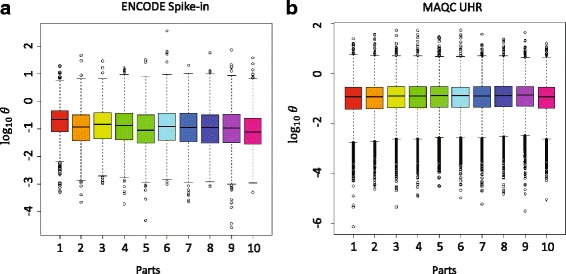
The pattern of overdispersion on parts of genes. The overdispersion rate was estimated on any position in 10 categories with equal data points according to the distance to the end of the genes. Part 1 is located on the gene tail and Part 10 is located on gene start. **a** ENCODE spike-in dataset. **b** MAQC UHR dataset. For strand-specific sequencing, only reads generated with mate2 primers on antisense strand were investigated. x-axis shows categories from the end of the genes

Elements of the sequencing procedure (e.g., fragmentation methods, random hexamer priming, etc.) can introduce types of bias to RNA-seq measurements [12]. We compared the overdispersion rates estimated from two datasets with different RNA-seq protocols (described in Methods) in Fig. 3. Interestingly, in the ENCODE dataset, the overdispersion rates were significantly larger at the tail (less than ~200 base pairs) of the genes. The same result was obtained in the calculation of the variance (Additional file 1: Figure S1). This may suggest a bias in ENCODE dataset. Therefore, we removed the reads mapped to the last 200 base pairs of each gene in our analyses to avoid this extra bias. However, no such difference was observed in MAQC UHR datasets.

This discrepancy might be explained by the different processes in sequencing library preparation of these two studies. In the ENCODE study, fragment selection after cDNA PCR amplification might lead to a loss of many fragments located at the transcript tails, thereby introducing an additional error. By contrast, according to the protocol used in the MAQC study, fragmentation was carried out prior to cDNA PCR amplification, leading to the same process of selection across the entirety of the gene.

### Models of the Overdispersion rate

**Fig. 4:**
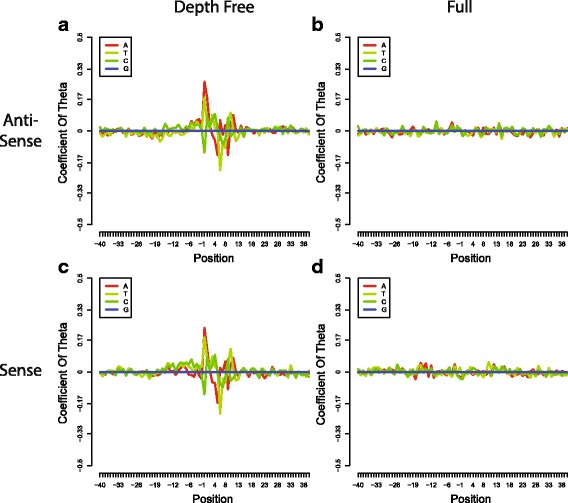
Coefficients of local sequence from the MAQC UHR dataset. x-axis shows the positions around the 5′ end of mapped reads, which was labelled as 0. Coefficients were calculated by two models on different strands: **a** Depth-free model on antisense strand, **b** Full model on antisense strand, **c** Depth-free model on sense strand and **d** Full model on sense strand

To reveal the confounding effects of the local primer sequence and the sequencing depth on the overdispersion rate, we studied two models: the full model with parameters for both the local primer sequencing and the sequencing depth and the depth-free model without parameters for the sequencing depth (described in Methods). After the linear formula transformation (Eq. 5), 240 coefficients of 80 positions around the primers were estimated efficiently. Coefficients estimated from MAQC UHR data were plotted against their corresponding positions in Fig. 4. From the depth-free model, we observed a similar pattern to those reported by Hansen et al. and Li et al. [12, 13] (Fig. 4a, c). However, no such pattern was observed from the full model (Fig. 4b, d). We observed similar results from the ENCODE spike-in data as well (Additional file 1: Figure S2). Both Hansen et al. and Li et al. demonstrated an association between hexamer primer and measurement count number. Plus, we observed in this study that the overdispersion rate on base pair decreased with increasing sequencing depth (Fig. 2). These findings lead to an inference that a hexamer primer might influence the overdispersion rate by affecting the count number; consequently, upon adjustment by count number, the relationship between the use of a hexamer primer and the overdispersion rate was no longer significant as observed in the full model (Fig. 4a, c). In addition, we calculated the coefficient of determination *R* ^2^ using a 5-fold cross-validation strategy (described in Methods). *R* ^2^ values of 0.481 and 0.488 were obtained for the depth-free model and the full model, respectively, from the MAQC UHR data; while values of 0.270 and 0.273, respectively, were obtained from the ENCODE spike-in data. Therefore, about half of the variance was explained by our models for the MAQC UHR dataset. Also, as expected, the depth-free model achieved a similar *R* ^2^ with the full model.

We investigated the influence of primers corresponding to the reads from the antisense and sense strands, respectively. We observed from the MAQC UHR dataset that reads mapped to antisense and sense strands showed quite similar patterns (Fig. 4a, c), which was consistent with the finding of Hansen et al. [12]. However, the reads on the sense strand should not be primer-related because they were synthesized by the RNase H niche method without hexamer priming. Hansen et al. [12] explained that the hexamer primer might not be completely digested. In contrast, this dependency was not observed on sense strands in the ENCODE spike-in dataset (Additional file 1: Figure S2). Its strand-specific protocol might be responsible for the different patterns on two strands, but further validation studies are required. In the present study, we estimated coefficients of local sequence separately for each strand in the present study.

### Comparison of four models

#### Goodness of fit

**Fig. 5:**
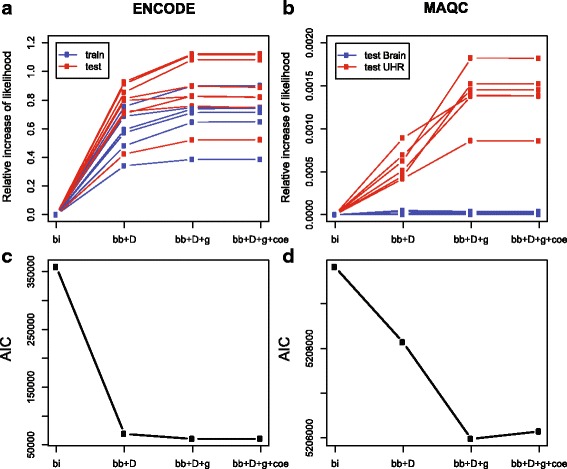
Goodness-of-fit on pairwise comparison of replicates. **a**, **c** ENCODE dataset. **b**, **d** MAQC dataset. **a**, **b** The mean percentages of change in the likelihood value compared to the nested model. **c**, **d** The mean AICs measured for four models

Comparing likelihood values is a straightforward way to select statistical models. We calculated likelihood values from four models: bi, bb + D, bb + D + g and bb + D + g + coe (described in Methods). As expected, the models with additional parameters had higher maximum likelihood values. Figure 5a shows the increase of likelihood value of the ENCODE spike-in dataset. The bb + D model made a huge jump from the bi model (improved by 30% - 90%, Chi-square test *p*-value <0.001). And the parameter *γ* in dynamic *θ*_*ij*_ in bb + D + g model also improved the fit by roughly 15% (Chi-square test *p*-value <0.001). However, the full model had no significant improvement from the primer-free model (Chi-square test *p*-value = 1), and the latter had the lowest AIC (Fig. 5c). We observed similar results in both training and test datasets and from the MAQC dataset as well (Fig. 5b, d; results for training dataset not shown). However, due to the small experimental library effect in the MAQC UHR dataset [7], increase of likelihood was not as significant as that shown in the ENCODE dataset. As expected, no difference of data fit was observed on MAQC brain samples which were from the same library (Fig. 5b).

#### DE detection

**Fig. 6:**
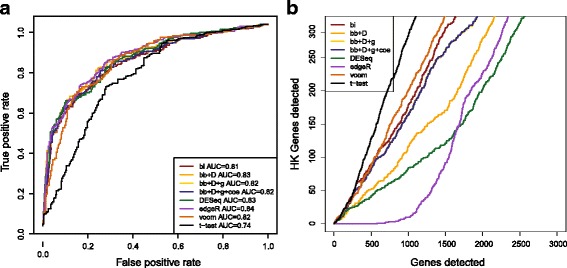
Performance of DE detection. DE genes were detected by comparing MAQC Brain samples to UHR samples. 7 methods (bb + D, bb + D + g, bb + D + coe, *t* test on logarithm transformed RNA-seq read counts, DESeq, edgeR and limma-voom) were applied. **a** ROC and **b** false housekeeping gene detections were used to evaluate their performances

**Fig. 7:**
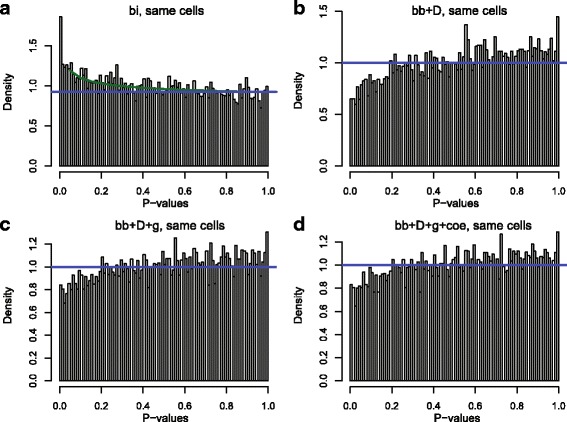
Histograms of *p*-values from comparison of replicates. *p*-values were calculated by **a** binomial model, **b** beta-binomial model with constant *θ*_*ij*_, **c** the primer-free beta-binomial model and **d** the full beta-binomial model. *Blue* line indicates an estimated uniform distributions; *green* line indicates a mixture distribution of beta distribution and uniform distribution

Further, we compared the AUC of DE analysis performance based on four models (bi, bb + D, bb + D + g, bb + D + coe) and widely used methods including *t* test on logarithm transformed RNA-seq read counts, DESeq, edgeR and limma-voom (Fig. 6a). As a result of the small library effect, no significant difference was observed between these four binomial based models when comparing MAQC brain and UHR samples, which agreed with our previous gene level study [7]. However, our beta-binomial based models (bb + D, bb + D + g, bb + D + coe) had good performances close to DESeq, edgeR, and limma-voom, which are slightly better than binomial-test and significantly superior to Student’s *t* test. Similar results were observed on the false discovery control, but DESeq, edgeR and bb + D falsely identified the least number of housekeeping genes given a certain number of discoveries (Fig. 6b). Testing the different library preparations from a same sample, bb + D produced non-uniformly distributed *p*-values with insufficient small ones (Fig. 7b), whereas bi had an overabundance of small *p*-values (Fig. 7a). In contrast, the histogram of the *p-*values was more flat for the beta-binomial models with a dynamic overdispersion rate, bb + D + g and bb + D + g + coe (Fig. 7c, d), indicating that the errors between samples from different libraries were captured more accurately by these two models.

## Discussion

In this study, we accurately modeled of the non-uniformity of RNA-seq read counts at the base level. We investigated the relationship of overdispersion rate with sequencing depth, local sequence, and library preparation protocols to study the properties of overdispersion. Based on these properties, base-level models are proposed to estimate the overdispersion rate accurately.

To the best of our knowledge, this is the first study of the confounding effects from sequencing depth and local sequence on overdispersion rate. We found they are strongly associated with each other. First, the overdispersion rate decreases as the sequencing depth increases on the base level. Second, random hexamer priming can notably influence the overdispersion rate. However, with the count number as a covariate in the modeling, the local sequence showed little influence on the overdispersion rate. Consequently, it is preferable to use the primer-free model with less parameters for superior computing efficiency and power.

Together with various systematic errors that have been identified in differential RNA-seq protocols and platforms [30, 31], our new findings provide important insights into the development of bias correction strategies in RNA-seq analyses. Based on the observation of extra noise on the tails of transcripts when fragmentation was performed before PCR, we concluded that experimental protocols before sequencing may influence the overdispersion rate of the RNA-seq reads and that the order of steps in the protocol matters. Therefore, we suggest removing the last 200 base pairs if fragmentation is performed before PCR in RNA-seq library preparation. Moreover, we suggest further studies of RNA-seq non-uniformity on sense and antisense strands separately.

Compared with models which ignore the overdispersion rate or use a constant overdispersion rate, bb + D + g accounting for a dynamic overdispersion rate fits the RNA-seq counts best with the highest likelihood value and the lowest AIC. It produced a similar AUC to popular DE analysis methods including DESeq, edgeR and limma-voom. bb + D showed the best false discovery control among proposed models, which may result from its insufficient power to detect small alterations (Fig. 7b). Theoretically, our model has two main advantages compared to these widely used DE analysis tools: (1) the catastrophe-resistant ability. The gene-level read counts might be susceptible to positions with high counts but with high fluctuations. Our model addresses this issue by down-weighting those unreliable read counts with highly variable dispersion rate and (2) borrowing information from spike-in measurement. Usually few experimental replicates are performed due to the cost. Spike-in transcripts, measured along with the samples, can be used a cost-effective alternative to estimate overdispersion rate.

The current study investigated the dependency between the overdispersion rate and the sequencing depth using replicates with no biological variance. However, the relationship between replicates with biological variance and systematic effect remains elusive. SEQC dataset, which was specifically designed to test the intra- and inter-site reproducibility [22, 32], warrants the future studies of that relation in the context of systematic effects. Also, the current model can be used to detect any base-level changes including gene expression alteration and differential exon usage. The exon level or isoform level differential analysis is thus required to take different usage of exons between samples into consideration.

## Conclusions

In conclusion, the current study provides thorough insights into the property of the overdispersion rate on the position level, especially into its relationship with sequencing depth, local sequence, and preparation protocol. These properties of RNA-seq will aid in improvement of quality control procedures and the development of statistical methods for downstream RNA-seq data analyses. Based on these properties, we propose a method to model the non-uniformity measurement in comparison study. Still, new sequencing strategies and protocols are emerging rapidly, such as the PCR-free sequencing technique [33]. The properties of sequencing reads as well as the biases and effects vary among different platforms. Future studies on investigating these properties are necessary to improve the methods for modeling RNA-seq data.

## Additional file

Additional file 1: Figure S1. The pattern of variance on parts of genes. **Figure S2.** Coefficients of local sequence from the ENCODE dataset. (DOCX 423 kb)

## Abbreviations

AIC: Akaike information criterion
AUC: Area under the receiver operating characteristic curve
DE: Differential expression
ERCC: External RNA Control Consortium
PCR: Polymerase chain reaction
