# Supplementary material for: Local sequence and sequencing depth dependent accuracy of RNA-seq reads
Source: BMC Bioinformatics. 2017 Aug 9;18:364. doi: 10.1186/s12859-017-1780-z (PMC5550947; doi:10.1186/s12859-017-1780-z)
Supplement: Additional file 1: Figure S1. — The pattern of variance on parts of genes. Figure S2. Coefficients of local sequence from the ENCODE dataset. (DOCX 423 kb) [file 12859_2017_1780_MOESM1_ESM.docx]

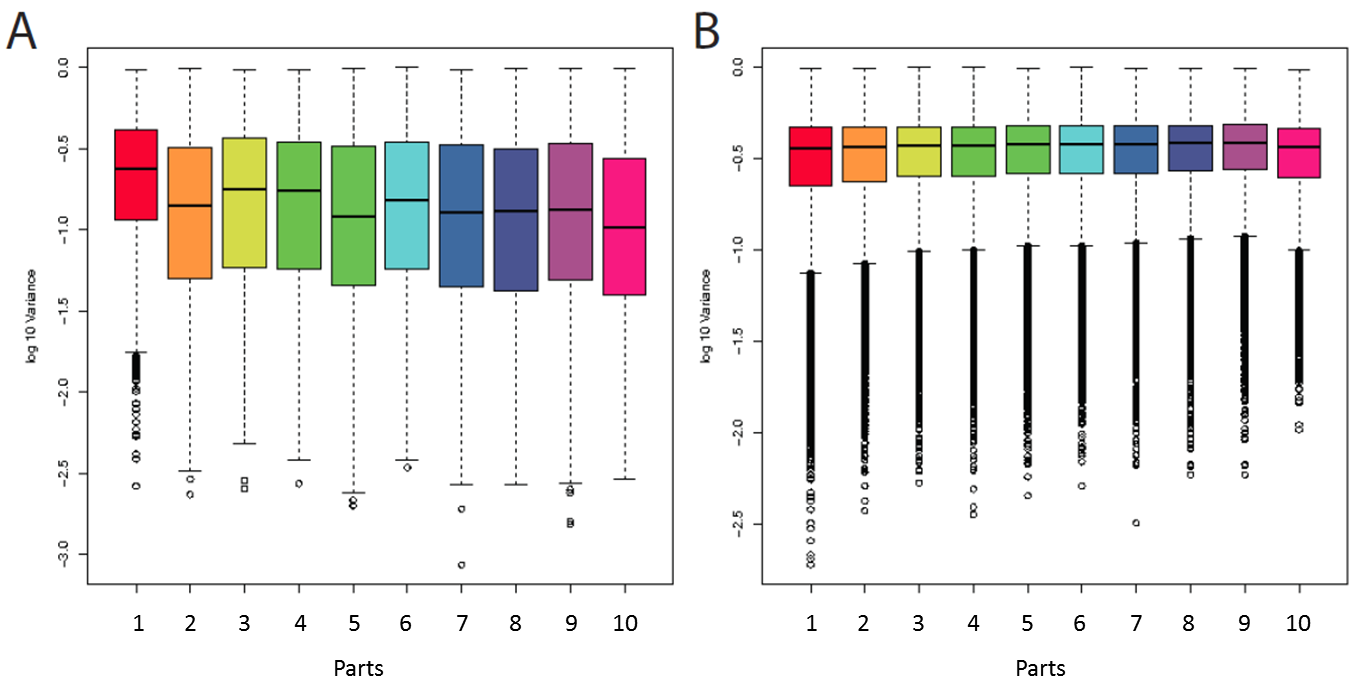


**Figure 1 - The pattern of variance on parts of genes.**

The variance estimated on any position in 10 equal categories according to the distance to the end of the gene. Part 1 is located on the gene tails and Part 10 is located on gene starts. (A) ENCODE spike-in dataset. (B) MAQC UHR dataset. Only mate2 on the antisense strand were investigated.


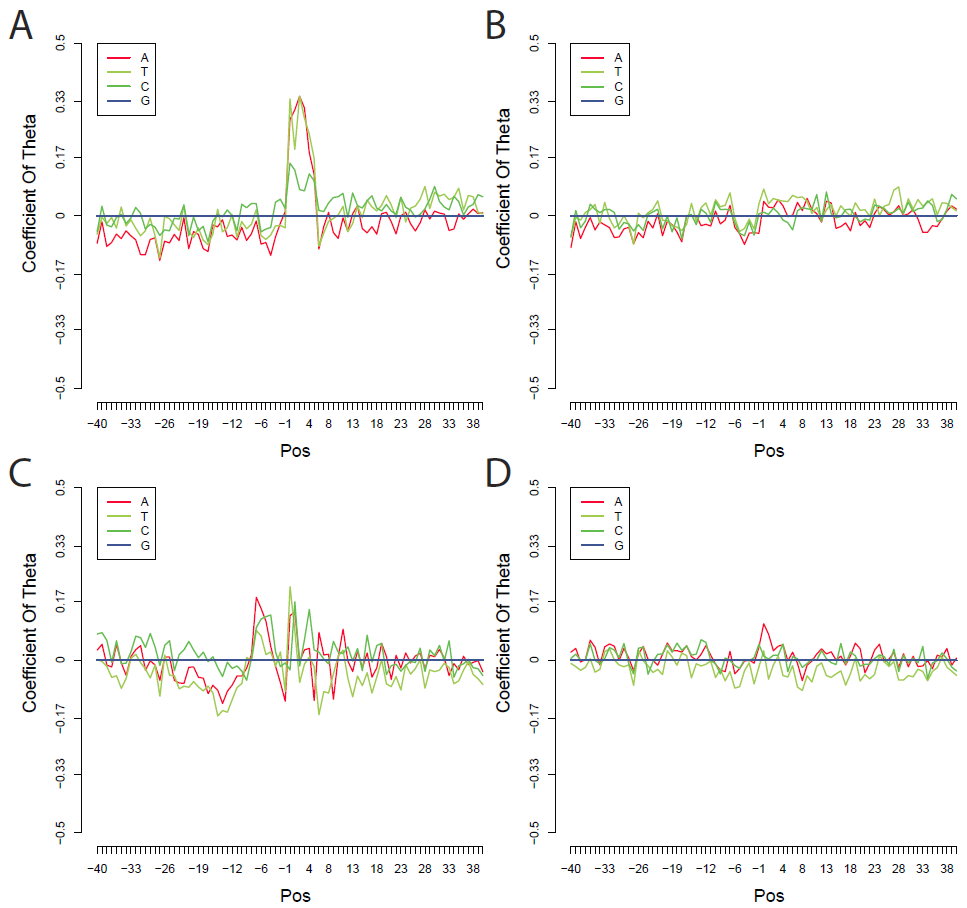


**Figure 2 - Coefficients of local sequence from the ENCODE dataset.**

x-axis shows the positions around the 5’ end of mapped reads which was labelled 0. Coefficients were calculated by two models. (A) Depth-free model on antisense strand. (B) Full model on antisense strand. (C) Depth-free model on sense strand. (D) Full model on sense strand.
